# Metabolomic, photoprotective, and photosynthetic acclimatory responses to post‐flowering drought in sorghum

**DOI:** 10.1002/pld3.545

**Published:** 2023-11-13

**Authors:** Christopher R. Baker, Dhruv Patel‐Tupper, Benjamin J. Cole, Lindsey G. Ching, Oliver Dautermann, Armen C. Kelikian, Cayci Allison, Julie Pedraza, Julie Sievert, Aivett Bilbao, Joon‐Yong Lee, Young‐Mo Kim, Jennifer E. Kyle, Kent J. Bloodsworth, Vanessa Paurus, Kim K. Hixson, Robert Hutmacher, Jeffery Dahlberg, Peggy G. Lemaux, Krishna K. Niyogi

**Affiliations:** ^1^ Howard Hughes Medical Institute, Department of Plant and Microbial Biology University of California Berkeley California USA; ^2^ Department of Plant and Microbial Biology University of California Berkeley California USA; ^3^ DOE‐Joint Genome Institute Lawrence Berkeley National Laboratory Berkeley California USA; ^4^ UC‐ANR Kearney Agricultural Research and Extension (KARE) Center Parlier California USA; ^5^ Environmental Molecular Sciences Laboratory, Pacific Northwest National Laboratory Richland Washington USA; ^6^ Biological Sciences Division, Pacific Northwest National Laboratory Richland Washington USA; ^7^ Department of Plant Sciences University of California Davis California USA; ^8^ Molecular Biophysics and Integrated Bioimaging Division, Lawrence Berkeley National Laboratory Berkeley California USA

**Keywords:** antioxidants, drought tolerance, galactinol, metabolomics, photoprotection, photosynthesis, *Sorghum bicolor*, stay‐green, stomatal closure

## Abstract

Climate change is globally affecting rainfall patterns, necessitating the improvement of drought tolerance in crops. *Sorghum bicolor* is a relatively drought‐tolerant cereal. Functional stay‐green sorghum genotypes can maintain green leaf area and efficient grain filling during terminal post‐flowering water deprivation, a period of ~10 weeks. To obtain molecular insights into these characteristics, two drought‐tolerant genotypes, BTx642 and RTx430, were grown in replicated control and terminal post‐flowering drought field plots in California's Central Valley. Photosynthetic, photoprotective, and water dynamics traits were quantified and correlated with metabolomic data collected from leaves, stems, and roots at multiple timepoints during control and drought conditions. Physiological and metabolomic data were then compared to longitudinal RNA sequencing data collected from these two genotypes. The unique metabolic and transcriptomic response to post‐flowering drought in sorghum supports a role for the metabolite galactinol in controlling photosynthetic activity through regulating stomatal closure in post‐flowering drought. Additionally, in the functional stay‐green genotype BTx642, photoprotective responses were specifically induced in post‐flowering drought, supporting a role for photoprotection in the molecular response associated with the functional stay‐green trait. From these insights, new pathways are identified that can be targeted to maximize yields under growth conditions with limited water.

## 1 INTRODUCTION

Worldwide, drought remains the primary abiotic cause of agricultural yield loss, and climate change may accelerate the impact of drought on agriculture as the frequency and severity of droughts increase (Lesk et al., 2016). The overuse of groundwater, largely driven by agricultural demand (Giordano, 2009; Giordano et al., 2019), also limits irrigation as a long‐term solution to maintaining agricultural productivity in a world experiencing hotter temperatures (Lobell et al., 2014; Ort & Long, 2014). Defining and tweaking the molecular mechanisms underlying drought‐adaptation traits in plants is vital to maintaining high yields under expected future climatic conditions (Varshney et al., 2018).

Drought tolerance is a complex, quantitative trait dependent on plant developmental stage and the severity of the water deficit (Luo et al., 2019). Crops, like sorghum, that perform C4 photosynthesis, an evolutionary innovation in the carbon (C) reactions of photosynthesis and anatomy of the leaf tissue, have increased intrinsic water‐use efficiency (*WUE*_*i*_) relative to crops that use C3 photosynthesis (Jones, 1992). Of the C4 crops, sorghum [*Sorghum bicolor* (L.) Moench] is exceptionally drought tolerant (Kimber, 2000), and the timing of drought before anthesis (pre‐flowering drought) or post‐anthesis (post‐flowering drought) has markedly different outcomes (Rosenow et al., 1996; Rosenow & Clark, 1995; Varoquaux et al., 2019). In the case of post‐flowering drought stress, stalk‐lodging rates and leaf senescence can increase, and grain size and grain yield can decrease (Thomas & Howarth, 2000).

The extent of post‐flowering drought tolerance also differs between sorghum genotypes, with so‐called “stay‐green” genotypes able to delay the senescence of the upper canopy until after the final stages of grain filling (Borrell et al., 2000; Krieg & Hutmacher, 1986). In “functional stay‐green” plants, such as the sorghum genotype BTx642, delayed leaf senescence in terminal post‐anthesis water deprivation is part of a suite of advantageous traits contributing to maintenance of high grain yields and grain size and prevention of stalk lodging (Harris et al., 2007; Thomas & Howarth, 2000; Tuinstra et al., 1997). In contrast, so‐called “cosmetic stay‐green” plants block chlorophyll degradation and, thus, remain green in drought but do not maintain high yields (Hörtensteiner & Kräutler, 2011; Thomas & Howarth, 2000).

At the whole‐plant level at anthesis, BTx642 has less tillering and less above‐ground biomass per plant relative to post‐flowering drought‐susceptible sorghum genotypes (Borrell, Mullet, et al., 2014; Borrell, van Oosterom, et al., 2014). At the cellular level, stay‐green sorghum genotypes maintain the integrity of the photosynthetic machinery through the grain‐filling period in post‐flowering drought (Borrell et al., 2001; Varoquaux et al., 2019). An additional important point is that maintenance of photosynthetic leaf area during post‐flowering drought will only be beneficial to the genotype if sufficient water reserves are available to allow stomata to remain partly open for CO_2_ assimilation (Borrell et al., 2001; Kamal et al., 2019; Varoquaux et al., 2019).

Leaf senescence during drought can be induced by elevated reactive oxygen species (ROS) levels (Cruz de Carvalho, 2008; Noctor et al., 2014). Excess excitation energy in drought drives ROS production, leading to the peroxidation of polyunsaturated lipids, damage to proteins, and the inactivation of pigments and antioxidants. Plants have evolved a suite of photoprotective responses to manage ROS (Li et al., 2009). These include photoprotective antioxidants in photosynthetic and epidermal tissues, such as ascorbate, tocopherols, and photoprotective flavonoids (Agati & Tattini, 2010; Li et al., 2009; Logan et al., 2006), as well as activation of non‐photochemical quenching (NPQ), the controlled dissipation of excess excitation energy as heat (Cousins et al., 2002; Golding & Johnson, 2003; Jung, 2004; Lima Neto et al., 2017; Ogbaga et al., 2014). Thus, strong photoprotective responses may act as a key post‐flowering drought tolerance trait; however, direct evidence is lacking for this hypothesis.

The molecular responses of sorghum to post‐flowering drought in the field have not been extensively characterized. As a first step, the time‐resolved transcriptomic response was determined for pre‐flowering and post‐flowering droughted field‐grown sorghum genotypes, BTx642 and RTx430 (Varoquaux et al., 2019). Paralleling this study, these two drought‐tolerant genotypes were grown in replicated, irrigated plots in the California Central Valley in 2019 under both control and post‐flowering drought conditions. BTx642 was selected as a functional stay‐green variety, whereas RTx430 has strong drought tolerance but lacks the full suite of stay‐green traits (Crasta et al., 1999). A third genotype planted in this field trial, RTx7000, was ultimately excluded from our study due to insufficient seed germination rates. There were two goals of this study: (1) determination of which metabolites may act as regulators of photosynthetic performance via controlling stomatal behavior in post‐flowering drought in the field and (2) testing whether the exceptional capacity of the stay‐green genotypes to maintain photosynthetic activity in post‐flowering drought involved the stronger activation of photoprotective responses relative to non‐stay‐green varieties. Photosynthetic, photoprotective, and water dynamics traits under control and droughted field growth conditions were quantified in both genotypes across multiple drought timepoints, and samples were harvested for metabolomic and lipidomic analysis. Physiological and metabolomic datasets were then compared with transcriptomic data collected from the same genotypes using the same growth regime in a prior year (Varoquaux et al., 2019). We find a robust correlation between foliar galactinol levels and stomatal response to post‐flowering drought, and we confirm our hypothesis that photoprotective responses are more strongly induced in a functional stay‐green sorghum genotype.

## 2 MATERIALS AND METHODS

### 2.1 Field growth and irrigation conditions

**FIGURE 1:**
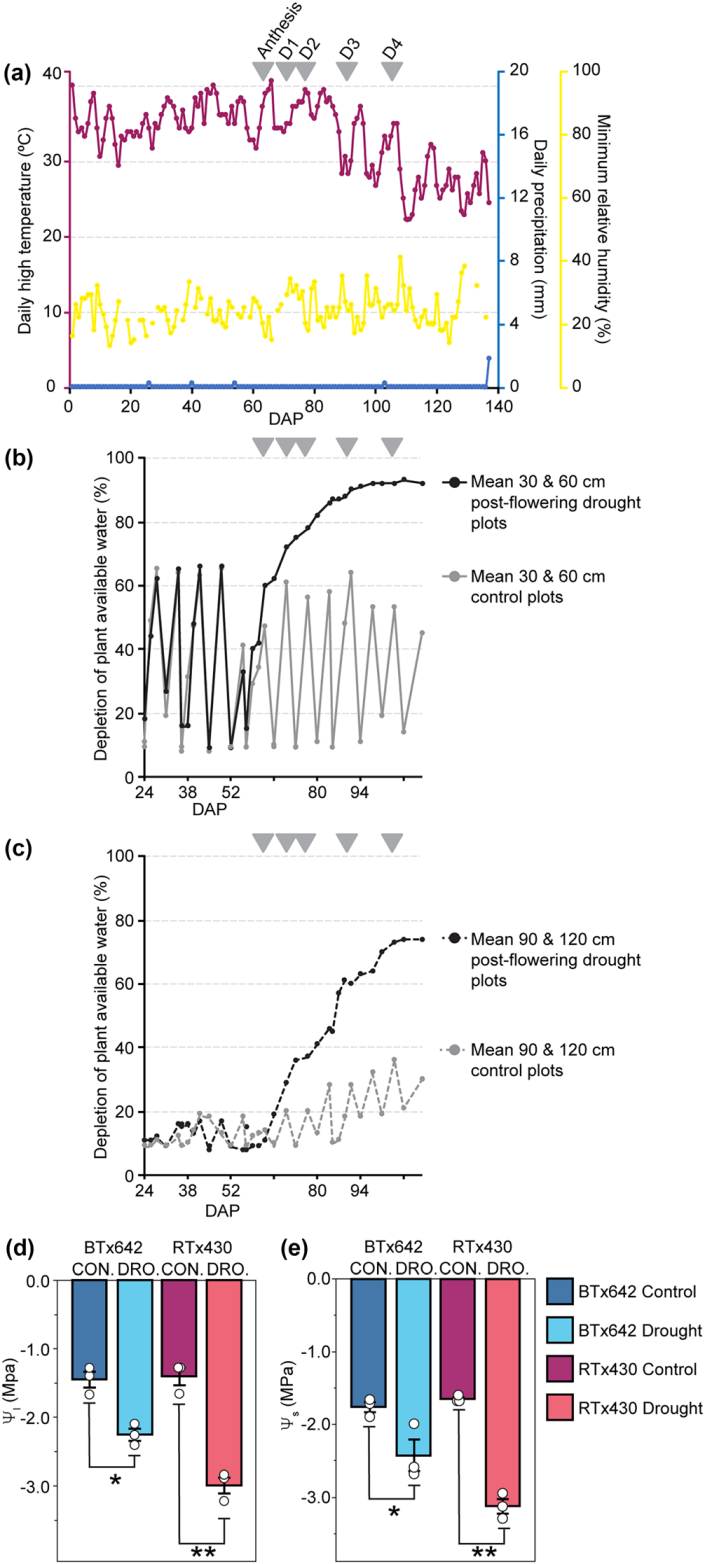
Field conditions, soil water depletion, and leaf water potential response to terminal drought stress. (a) Data collected from June 11 to October 26, 2019, at Parlier Weather Station A (Parlier, CA, USA). Daily high temperature (axis 1, magenta), daily precipitation (axis 2, blue), and minimum relative humidity (axis 3, yellow). The *y*‐axis upper bound for each variable is set to the daily annual maximum value for 2019. (B,C) Soil water data expressed as percent plant available soil water depletion between −.02 MPa (field capacity) and −1.5 MPa (permanent wilting point). Drought plots (dark gray) and control plots (light gray) with sensors at (b) 30 and 60 cm (solid lines) and (c) 90 and 120‐cm depth (dashed lines). Sampling dates are labeled as D1 through D4. (d) Midday leaf water potential (Ψ_I_) and (e) osmotic potential (Ψ_S_) collected on D4 (40 days without water). BTx642 control (dark blue), BTx642 drought (light blue), RTx430 control (purple), and RTx430 drought (pink). Mean values ± standard errors (*n* = 3 plots) with mean values for each individual plot displayed as dots (white). Significant differences as measured by a two‐tailed *t* test for control versus treatment pairs are indicated by asterisks (* < .05, ** < .005).

Sorghum genotypes BTx642 and RTx430 were grown in Parlier, CA (36.6008°N, 119.5109°W) in 2019 in a Hanford sandy loam soil (pH = 7.37) with a silky substratum in .071‐ha plots of 10 rows each. Plots were arranged in a randomized complete block design (RCBD) with three replications of each genotype and water regime (Figure S1). Two watering conditions were used on plots: (1) control, consisting of weekly watering, based on evapotranspiration, 5 days prior to sampling dates, with the first irrigation starting 18 days after planting (DAP) and continuing until 123 DAP and (2) post‐flowering drought, consisting of regular irrigation up through and including irrigation at 65 DAP—at which point over 50% of the plants flowered (anthesis)—with terminal water deprivation from that point onwards (Figure 1). Pre‐planting irrigation was performed for all plots such that the upper 122 cm of soil would have been refilled to the field capacity. Following that, plots receiving water were irrigated at 7‐day intervals using drip irrigation lines placed on the soil surface of each furrow.

All irrigated plots received volumes of water equal to 100% of the average weekly calculated crop evapotranspiration for the 7‐day period before irrigation. Surface drip lines were used for irrigation to provide accurate water application amounts and a high level of water application uniformity. Irrigation once per week in plots is comparable with sorghum farming irrigation practices in the Western United States. Additionally, providing equal water volume to all irrigated plots prevents a scenario where genotypic differences in evapotranspiration rates lead to a difference in total water volume supplied to specific plots. For greater details on crop evapotranspiration and irrigation management, see the Supporting Information and Xu et al., (2018).

Total final biomass was comparable between control plots for both genotypes with an average forage yield (65% moisture) of 32.17.02 T ha^−1^ for BTx642 and 32.82 T ha^−1^ for RTx430. Planting in 2019 occurred on June 10. Four sampling dates were selected, and for each date, control plots had not received water for 5 days: (1) August 20, 2019 (D1), 5 days since last watering for all plots, 70 DAP; (2) August 27, 2019 (D2), 12 days of post‐flowering drought, 77 DAP; (3) September 10, 2019 (D3), 26 days of post‐flowering drought, 91 DAP; (4) September 24, 2019 (D4), 40 days of post‐flowering drought, 105 DAP.

### 2.2 Leaf phenotypic traits

On each of the four sampling dates, gas exchange and chlorophyll fluorescence measurements were collected within two time windows: 9:30 to 11:00 (morning) and 14:00 to 16:00 (mid‐afternoon), using LI‐COR 6400XT instruments (LI‐COR, Lincoln, NE, USA). Given that these sampling dates all occurred post‐anthesis, all leaves had emerged, and thus, it was possible to randomly sample the uppermost three leaves including the flag leaf from plants growing in the interior of each plot on each sampling date. Each of the LI‐COR 6400XT instruments was factory calibrated the month prior to this field work, and the calibrations and instrument checks, as described in Chapter 4 of the LI‐COR 6400 manual, were performed on each sampling date. Leaves were maintained near ambient light levels and temperatures by measuring ambient PAR levels and local temperatures and re‐adjusting actinic light levels and blocking temperature prior to each set of measurements. The ratio of blue‐to‐red LED contribution to the cuvette light source was 10%/90%. Relative humidity in the measurement cuvette was maintained between 50% and 60% to maintain stomatal aperture width. Flow rate was set to 400 μmol s^−1^ and sample [CO_2_] to 400 μmol mol^−1^. Stability variables typically converged within 60 s of clamping a leaf, then an infrared gas analyzer match was performed, and once stability variables were restored following the match, the measurement was taken. Leaves were clamped to avoid the midrib and always near the midpoint of the leaf (i.e., equal distance from the tip and leaf base). A multiphase flash routine was used to estimate chlorophyll fluorescence parameters (Loriaux et al., 2013). Prior to the measurement of F_o_՛, a far‐red light pulse of 25‐μmol photons m^−2^ s^−1^ for 1 s was performed prior to activation of the actinic light and then the far‐red pulse was performed again for an additional 5 s, finally, ending 1 s prior to the measurement. A minimum of eight leaves were randomly sampled per plot per timepoint.

On 105 DAP, green leaf area images were collected, and F_v_/F_m_ and NPQ were determined. Specific to NPQ measurements, these values were measured exclusively on leaves without visible signs of leaf senescence in both control and droughted plots. This decision was made to ensure that photoprotective traits could be accurately quantified in leaves with photosynthetic machinery intact prior to the onset of leaf senescence traits. Green leaf area was determined by imaging the three uppermost leaves including the flag leaf on 10 randomly selected plants per plot. Stomatal density and guard cell length were quantified using leaf peels collected on the D4 sampling date from the abaxial leaf surface of the uppermost non‐flag leaf of the main culm (Lopez et al., 2017). More details of leaf phenotypic measurements can be found in the Supporting Information.

### 2.3 Sample collection and processing

Plant samples were collected manually on the same days as physiological measurements with root systems to a depth of approximately 30 cm. Three plants from each plot were collected, and the uppermost three leaves, stems (below the peduncle and above the node for the next leaf below), and roots were harvested to create a single leaf, stem, and root sample for each plot for each timepoint. Root tissue was collected as previously described (Xu et al., 2018). Root tissue collection avoided brace roots and consisted almost entirely of mature, differentiated roots, avoiding root tips to make this sample more comparable with the mature leaf tissue. After collection, roots were vortexed in epiphyte removal buffer (.75% KH_2_PO_4_, .95% K_2_HPO_4_, 1% Triton X‐100 in ddH2O; filter sterilized at .2 μm) for 5 min. All samples were then flash‐frozen in liquid nitrogen within 5 min of being removed from the field. Each week, all samples were collected less than 1 h after dawn (dawn), within 1 h of the midpoint of the light period (midday), and less than 1 h before dusk (dusk).

### 2.4 Metabolite extraction, quantification, and metabolomics

For details of metabolite extractions and spectrophotometric quantification of specific metabolites, see the Supporting Information. Leaf tissue samples from sampling dates D2, D3, and D4 were analyzed by gas chromatography–mass spectrometry (GC–MS), lipidomics, and solid‐phase extraction with ion mobility phase and mass spectrometry (SPE‐IMS‐MS). Metabolomic data were collected for stem and root samples from D2, D3, and D4 sampling dates exclusively by IMS. For GC–MS, MPLEx extraction was applied to the samples that were weighed at 1 g (Nakayasu et al., 2016). Then, samples were completely dried under a speed vacuum concentrator. Dried metabolites were chemically derivatized and analyzed as reported previously (Kim et al., 2015) and further described in the Supporting Information. Metabolites were initially identified by matching experimental spectra to an augmented version of the Agilent Fiehn Metabolomics Library, containing spectra and validated retention indices for almost 1000 metabolites (Kind et al., 2009) and additionally cross‐checked by matching with NIST17 GC/MS Spectral Library and Wiley Registry 11th edition. All metabolite identifications were manually validated to minimize deconvolution and identification errors during the automated data processing. Data were log_2_ transformed and then mean‐centered across the log_2_ distribution. C and N values were determined at the Center for Stable Isotope Biogeochemistry at UC‐Berkeley using leaf samples from the D4 time point. Organic nitrogen (*N*_*org*_) values were calculated by subtracting total N levels by spectrophotometrically determined ammonium (Ammonia assay kit, Megazyme, Bray, Ireland) and nitrate levels (Bloom et al., 2014).

For lipidomics, total lipid extracts (TLEs) were analyzed as outlined in Kyle et al. (2017) and further detailed in the Supporting Information.

For SPE‐IMS‐MS metabolomics, extracts were analyzed using a RapidFire 365 (Zhang et al., 2016) coupled with an Agilent 6560 Ion Mobility QTOF MS system (Agilent Technologies, Santa Clara, CA, USA) as described in detail in the Supporting Information. The PNNL‐PreProcessor v2020.07.24 (https://omics.pnl.gov/software/pnnl-preprocessor) was used to generate new raw MS files (Agilent MassHunter “.d”) for each sample, run with all frames (ion mobility separations) summed into a single frame and applying 3‐point smoothing in the ion mobility dimension and noise filtering with a minimum intensity threshold of 20 counts. Details of the data processing and compound identification can be found in the Supporting Information.

### 2.5 Statistical analysis

All statistical analyses were performed using JMP Pro 16 software (JMP, Cary, NC, USA) and analysis of variance (ANOVA) used to analyze the effects of treatment and genotype, appropriate for the RCBD field layout. Prior to the analysis of gas exchange values, six measurements (out of the 462 measurements taken) with physiologically impossible *C*_*i*_ values (*C*_*i*_ values < 0 μMol CO_2_ mol^−1^ air) were removed from our datasets and attributed to either machine or user error.

### 2.6 Transcriptomic data processing and visualization

To generate expression plots for selected gene sets, we obtained normalized counts of *S. bicolor* genes mapped to a common reference (*S. bicolor* BTx623) and accompanying metadata from the EPICON field trial described previously (Varoquaux et al., 2019). Normalized counts were then summarized for control‐treated leaf samples for each genotype, week, and gene by taking the arithmetic mean (*n* = 1–3) and Log_2_‐transformation (with a pseudocount of 1). These values were subtracted from Log_2_‐transformed (plus a pseudocount of 1) normalized counts for each locus, genotype, day, and treatment from the EPICON dataset, to generate a control mean‐corrected dataset of gene expression for pre‐ and post‐flowering drought treatments. These values were then plotted as points, with loess‐smoothed values computed from these transformed data plotted as lines.

## 3 RESULTS

### 3.1 Longitudinal photosynthetic response of sorghum to terminal post‐flowering drought in the field

Sorghum plants were grown in irrigated, replicated plots with 10 rows in each plot (see Figure S1 for details of the field layout). BTx642 and RTx430 plants reached 50% inflorescence emergence (anthesis) by 69 and 71 DAP, respectively (Figure 1, Table S1). Before anthesis, the average maximum daily temperature was 35.5°C with a range from 29.4–40.0 °C for maximum daily temperatures (Figure 1a). Post‐anthesis temperatures declined with an average maximum daily temperature of 34.6°C with a range of 26.7–40.6°C for maximum daily temperatures throughout the grain‐filling period. Relative humidity was in general low with an average minimum daily value of 23.9% with a range of 13–35% from the time of germination to the end of the grain‐filling period (Figure 1a). No precipitation occurred during the growth lifecycle (Figure 1a).

Prior to 65 DAP, control and post‐flowering drought plots for both genotypes received an equal volume of water once per week, matched to average evapotranspiration rates across the entire field (Figure 1b,c; see Section 2). After 65 DAP, post‐flowering drought (hereafter, “drought”) plots were terminally water deprived (Figure 1b,c). From the 30‐ to 60‐cm depth, plant‐available water was 90% depleted by 92 DAP (27 days without water) in droughted plots. From 90‐ to 120‐cm depth, water depletion plateaued at ~75% at 105 DAP (40 days without water). Water‐deficit stress in droughted plots decreased leaf water and osmotic potentials in both genotypes (Figure 1d,e, Table S1). Nevertheless, grain yields, seed weights, and forage yields in these two drought‐tolerant genotypes were not significantly decreased in droughted plots in either genotype relative to control (Table S1).

**FIGURE 2:**
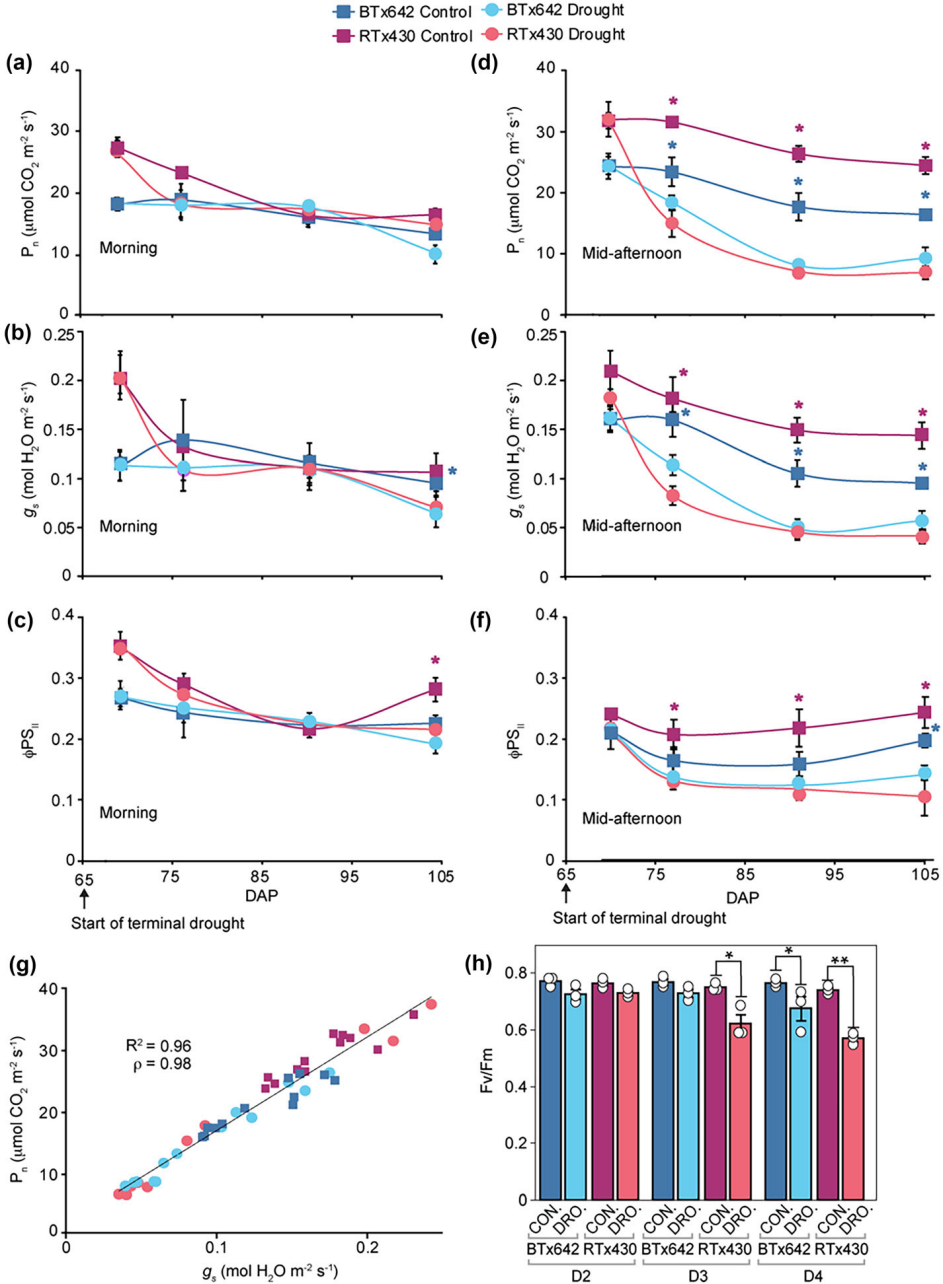
Photosynthetic response to terminal drought stress. (a–h) BTx642 control (dark blue), BTx642 drought (light blue), RTx430 control (purple), and RTx430 drought (pink). (a–c) Measurements in the morning (9:30 to 11:00) and (d–f) collected in mid‐afternoon (14:00 to 16:00 on the uppermost three leaves. (a,d) Net photosynthetic rate (*A*_*n*_), (b,e) stomatal conductance (*g*_*s*_), and (c,f) operating efficiency of photosystem II in the light (ΦPSII). Light levels ranged between 1651.3 and 1880.5 μmol photons m^−2^ s^−1^ for mid‐afternoon measurements and 1001.4–1199.4 μmol photons m^−2^ s^−1^ for morning measurements. *T*_*air*_ values for D1–D4 were 33.0°C, 39.3°C, 31.6°C, and 31.5°C, respectively, for mid‐afternoon measurements and 23.9°C, 28.1°C, 21.6°C, and 25.7°C, respectively, for morning measurements. Mean values ± standard errors (*n* = 3 plots). (a–f) Color of the asterisk denotes which control versus treatment pair has a *p* < .05 by a two‐tailed *t* test. (g) Linear curve fit to mid‐afternoon *g*_*s*_ and *A*_*n*_ values for each plot from D1–D4 timepoints with *R* ^2^ and Pearson correlation coefficient (ρ) values. (h) Maximum quantum efficiency of PSII (F_v_/F_m_) measured after 20 min of dark acclimation in the mid‐afternoon. Mean values ± standard errors (*n* = 3 plots) with mean values for each individual plot displayed as dots (white). Significant differences, as measured by a two‐tailed *t* test for control versus treatment pairs, are indicated by asterisks (* < .05, ** < .005).

Four sampling dates were selected that span the water depletion time‐course (sampling dates D1–D4, Figure 1a–c). The morning measurements (collected between 9:30 to 11:00) for net photosynthetic rates (*A*_*n*_), stomatal conductance (*g*_*s*_), and operating efficiency of PSII in the light (ΦPSII) revealed few statistically significant differences between control and droughted plots (Figure 2a–c). Two exceptions were a significant difference in *g*_*s*_ between control and drought in BTx642 and between control and drought in ΦPSII in RTx430 at D4 (105 DAP, 40 days without water, Figure 2b,c).

In contrast to morning measurements, drought repressed *A*_*n*_, *g*_*s*_, and ΦPSII in both genotypes in mid‐afternoon measurements (collected between 14:00 to 16:00) at D2 (77 DAP, 12 days without water), D3 (91 DAP, 26 days without water), and D4 (Figure 2d–f). *A*_*n*_ and *g*_*s*_ in droughted plots in the morning measurements were either higher or equal to mid‐afternoon measurements despite the higher photon flux density in the mid‐afternoon at D2, D3, and D4 (Figure 2a,d; see Table S2 for air temperatures and light levels on sampling dates).

In control plots, mid‐afternoon *A*_*n*_ and *g*_*s*_ were higher at all timepoints in RTx430 relative to BTx642 (Figure 2a,d). Consistent with its stay‐green phenotype, BTx642 extracted more soil water in post‐flowering drought plots relative to RTx430 (Table S3). We are confident in making phenotypic comparisons between genotypes grown in separate plots because (a) the total biomass (i.e., forage yield at 65% moisture, Table 1) was nearly equivalent between genotypes in control plots, (b) both genotypes received the same amount of water (see Section 2), and (c) the comparison between these genotypes grown under equivalent conditions has been made in other published works (Gao et al., 2019; Varoquaux et al., 2019; Xu et al., 2018).

**TABLE 1:** Linear fit for foliar molecules responding to drought in both genotypes.

		Correlation		Tests for non‐Normal distribution of residuals	
	Direction of response in drought	Correlation coefficient	Adjusted *p* value	Shapiro–Wilk	Anderson–Darling
Galactinol	+	.61	2.55E‐06	*n.s*.	*n.s*.
Maleic acid	−	.55	4.39E‐05	<.0001	<.0001
Fumaric acid	−	.54	4.33E‐05	.0001	<.0001
α‐Ketoglutaric acid	+	.32	3.64E‐02	.0001	.0008
PS	−	.2181582	*n.s*.	*n.d*.	*n.d*.

*Note*: Direction of response is positive (+) or negative (−) if molecule abundance was increased or decreased by drought treatment, respectively. Following line‐fitting, residuals were fit to a Normal curve and the Goodness‐of‐Fit was tested by Shapiro–Wilk and Anderson–Darling tests. A significant value for these tests demonstrates a non‐Normal distribution. For the *p* values reported in the final three columns, a significant value was considered *p* < .05 (*n.s*. = not significant, *n.d*. = not determined).

*A*_*n*_ and *g*_*s*_ were correlated for all mid‐afternoon measurements in both genotypes (Figure 2g). Along with stomatal closure, photoinhibition can contribute to the depression of *A*_*n*_ in moderate and severe drought. Dark‐acclimated maximum quantum efficiency of PSII (F_v_/F_m_) was not significantly depressed in droughted plants until D4 in the stay‐green genotype BTx642 and not until D3 in RTx430 (Figure 2h).

### 3.2 Galactinol abundance correlates with stomatal closure in field‐droughted sorghum

**FIGURE 3:**
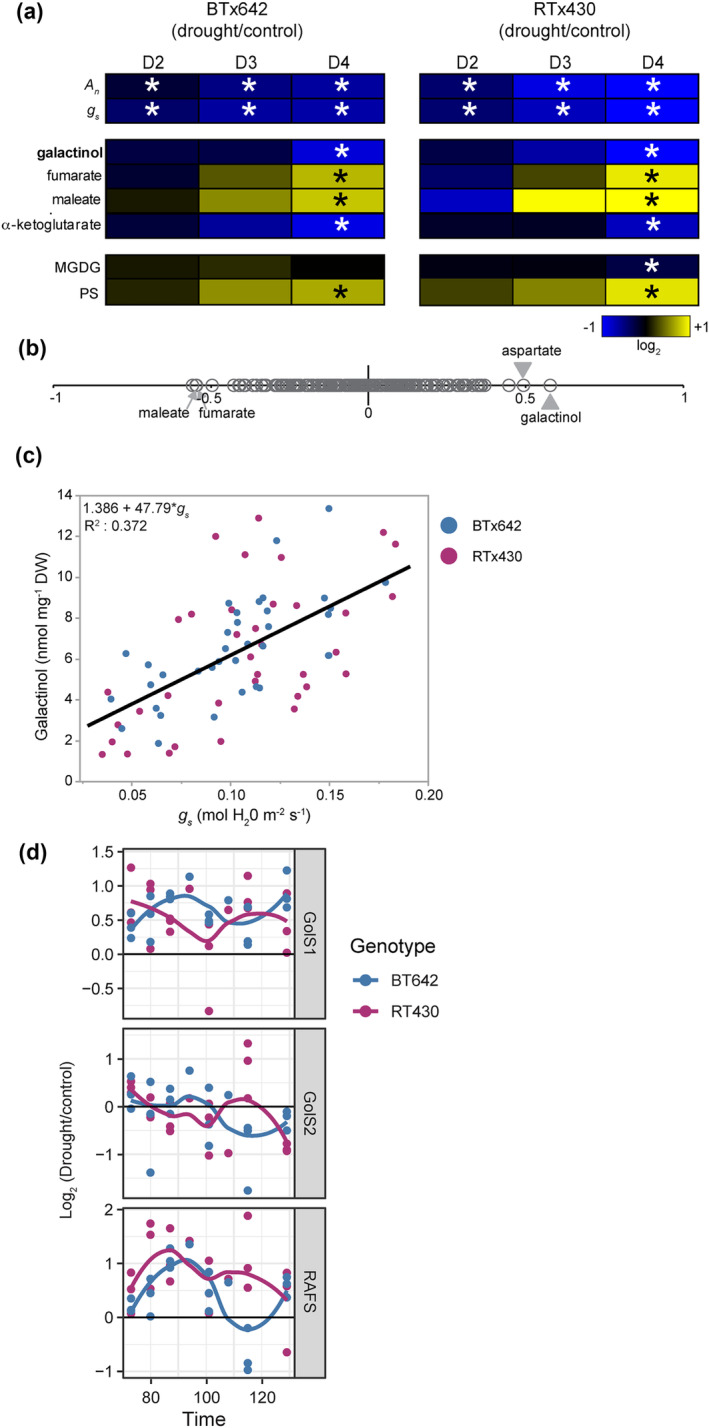
Metabolite and lipid abundance correlation with *g*_*s*_. (a) Differential abundance in leaf tissue for all metabolites/lipids that had a statistically significant response to drought in both genotypes at the same timepoint. Metabolite abundance in log_2_ scale with elevated concentration in drought (yellow) and decreased in drought (blue). Significant differences in abundance as determined by a two‐tailed *t* test with a Benjamini–Hochberg correction applied (adj. *p* < .05) in drought versus control are indicated by an asterisk. Black and white asterisks do not have separate meanings. In the top two rows, physiological data for *A*_*n*_ and *g*_*s*_ are included to assist visual comparison with patterns in the metabolite data. (b) Pearson correlation coefficients calculated for all 198 metabolites against *g*_*s*_. Positions of selected metabolites that had comparatively strong correlations with *g*_*s*_ are labeled. (c) Line fitting to *X*‐*Y* scatter of *g*_*s*_ and galactinol abundance data. In (c,d), BTx642 values are shown in blue, and RTx430 values are shown in purple. (d) Time‐course of log_2_ transcript abundance (drought/control) for post‐flowering drought timepoints for sorghum genes, galactinol synthases GolS1 (Sobic.001G391300) and GolS2 (Sobic.002G423600), and galactinol hydrolyase, RAFS (Sobic.003G052300).

The abundances of 198 metabolites were quantified in leaf, stem, and root tissue along with lipidomic sampling of 195 lipid species in leaf tissue at timepoints D2, D3, and D4. Once corrected for multisampling bias, only 34 of the 198 metabolites had a significant difference in abundance in response to drought in at least one genotype in leaf tissue (Figure S2, Table S4). Of these 34 metabolites, only four molecules responded significantly to drought in both genotypes at the same timepoint and in the same direction (Figure 3a). Thus, most drought‐induced changes in metabolite abundance were genotype‐specific. Among the metabolites that had a genotype‐specific response to drought were a number of potential osmoregulators. These included the increased levels of glucose, fructose, and *myo*‐inositol in BTx642, whereas RTx430 induced galactose and to a lesser extent fructose as well (Figure S2).

For the lipid data, the thylakoid lipid monogalactosyldiacylglyercol (MGDG) was specifically depressed in RTx430 at the final drought timepoint, whereas phosphotidylserine lipids (PS) were induced in both genotypes (Figure 3a).

Metabolite abundance was then correlated with *A*_*n*_ and *g*_*s*_ values measured in parallel with sample harvesting (Figure 3b). Data from both genotypes were analyzed together to increase the likelihood of finding patterns that were not genotype‐specific. For simplicity, only correlations with *g*_*s*_ are shown, given the tight correlation of *A*_*n*_ and *g*_*s*_ values (Figure 2g). Metabolites such as galactinol, α‐ketoglutarate, and aspartate shared a positive correlation with *g*_*s*_; their abundance declines with drought, whereas the organic acids fumarate and its isomer maleate had an inverse correlation, given their abundance increases with drought. Galactinol, maleate, fumarate, and α‐ketoglutarate were the only four molecules that had a statistically significant correlation with *g*_*s*_ after correcting for multiple sampling (Table 1, adj. *p*: <.05). Given the relatively weak correlations for some of these molecules, the Normality of the residuals from the line fit was tested for Goodness‐of‐Fit. If the residuals are not Normally distributed, this supports that the correlation may be superficially caused by outliers or only a small subset of the data. Indeed, the residuals for maleate, fumarate, and α‐ketoglutarate were, in fact, non‐Normally distributed when assessed for Goodness‐of‐Fit to a Normal distribution (Table 1, *p* < .05). Thus, out of all the metabolites and lipids measured, galactinol was the sole metabolite to have a robust correlation with *g*_*s*_ activity, as well as a statistically significant drought‐induced change in abundance in both genotypes even after correcting for multiple sampling bias (Figure 3a,c, Table 1).

Galactinol is synthesized by GolS enzymes, GolS1 (Sobic.001G391300), and GolS2 (Sobic.002G423600) (Figure 3d). In both genotypes, the transcript abundance of GolS1 is weakly induced post‐anthesis across the entirety of the post‐flowering drought period. In contrast, GolS2 transcript abundance trends downward across the post‐flowering drought period. This downward trend in GolS2 levels is consistent with the gradual decline in galactinol abundance in post‐flowering droughted plots. Galactinol is then consumed by the galactinol hydrolase SbRAFS (Sobic.003G052300). SbRAFS transcript abundance was induced throughout the post‐flowering drought period in both genotypes (Figure 3d).

### 3.3 Stronger photoprotective response minimizes photooxidative stress in BTx642

**FIGURE 4:**
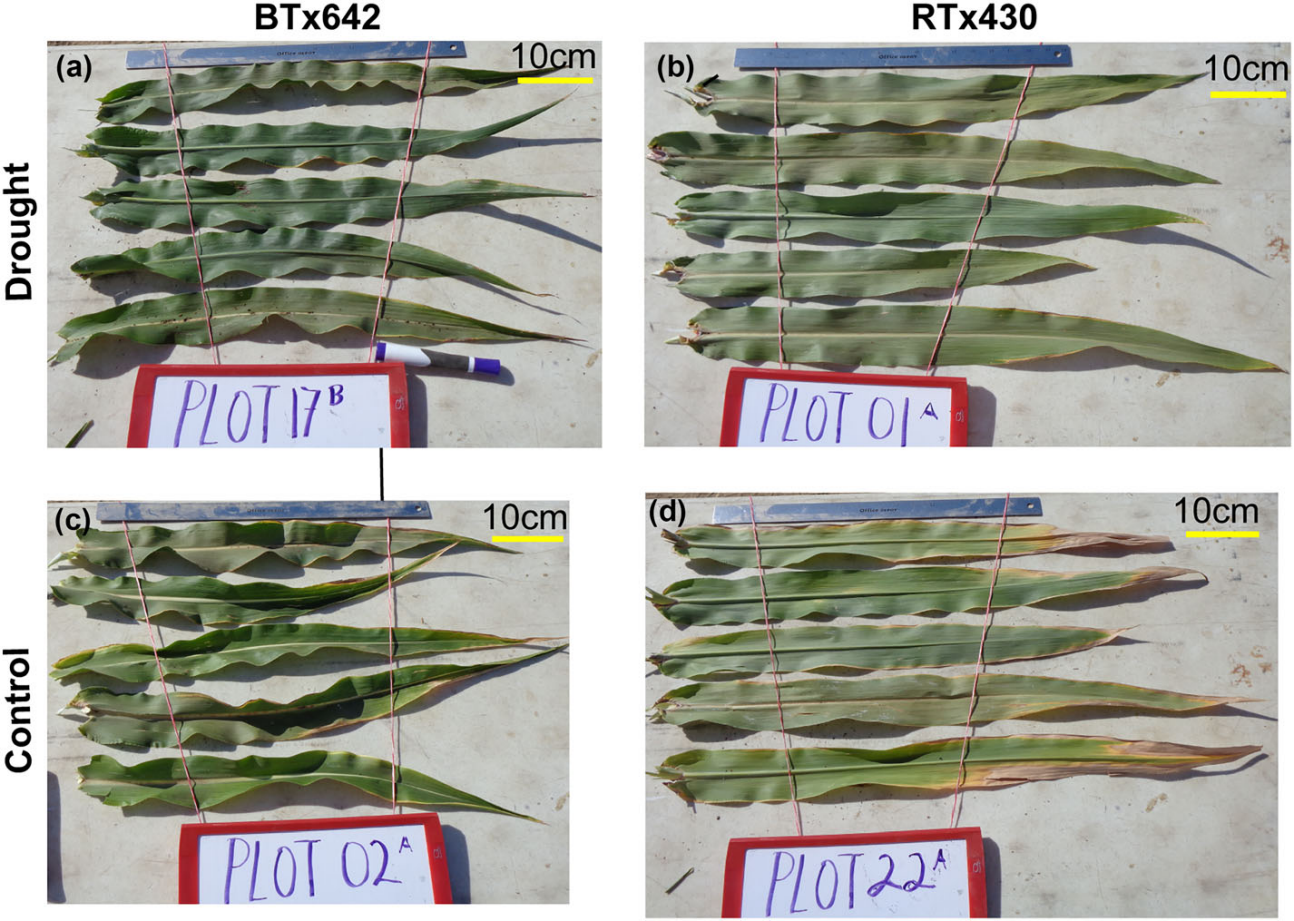
Representative photos of leaves in control and post‐flowering drought plots. Leaves sampled on D4 (40 days without water for droughted plots) from five different plants and a randomly selected leaf from the uppermost three leaves including flag leaves. (a) BTx642 control, (b) RTx430 control, (c) BTx642 post‐flowering drought, and (d) RTx430 post‐flowering drought.

To maintain photosynthetic leaf area in drought, plants must effectively manage photooxidative stress induced under drought conditions. However, the importance of photoprotective responses to the “stay‐green phenotype” in post‐flowering drought tolerance has not been examined. A two‐way ANOVA supports that *A*_*n*_, *g*_*s*_, and percent green leaf each had strong treatment and genotype × treatment effects (Figure 4, Table 2). For all three of these parameters, the net decline at the final D4 timepoint from control to drought had a larger magnitude in the RTx430 plots, consistent with the stay‐green phenotype of BTx642. Declines in foliar C/N and organic N (*N*_*org*_) content, often associated with a loss in green leaf area, were observed in both genotypes, but there was not a significant genotype × treatment effect for either of these parameters (Table 2).

**TABLE 2:** Two‐way ANOVA for gas exchange and leaf phenotypic traits.

Variable	BTx642			RTx430			Two‐way ANOVA		
	Control	Drought	Δ (drought − control)	Control	Drought	Δ (drought − control)	Genotype	Treatment	Genotype × treatment
*A*_*n*_ (μmol CO_2_ m^−2^ s^−1^)	16.4	9.25	−7.15	24.46	6.87	−17.59	***	*	***
*g*_*S*_ (mol H_2_0 m^−2^ s^−1^)	.095	.056	−.038	.14	.041	−.1	***	*	***
*WUE*_*i*_	6.42	5.65	−.76	6.71	4.86	−1.85	*	*n.s*.	*n.s*.
*R*_*d*_ (μmol CO_2_ m^−2^ s^−1^)	−4.99	−2.35	2.64	−3.97	−2.41	−1.55	***	*n.s*.	*n.s*.
Green leaf area (%)	95.8	85.18	−10.62	98.38	75.68	−22.7	***	*	***
C/N	17.6	22.85	5.25	18.47	24.04	5.57	***	*n.s*.	*n.s*.
*N*_*org*_ (mg/g DW^−1^)	24.1	18.86	−4.3	23.74	18.12	−4.19	***	*n.s*.	*n.s*.

*Note*: Significant *p* values determined using a two‐way ANOVA are denoted by asterisks.

Abbreviations: ANOVA, analysis of variance; *n.s*., not significant.

* *p* < .05,

** *p* < .005, and

*** *p* < .0005.

The greater maintenance of green leaf area in the upper canopy in BTx642 and, therefore, greater photosynthetic potential may depend on stronger photoprotective mechanisms in BTx642 preventing photoinhibition. Consistent with this hypothesis, F_v_/F_m_ in droughted RTx430 was lower at D3 and D4 relative to droughted BTx642 (Figure 3h).

**FIGURE 5:**
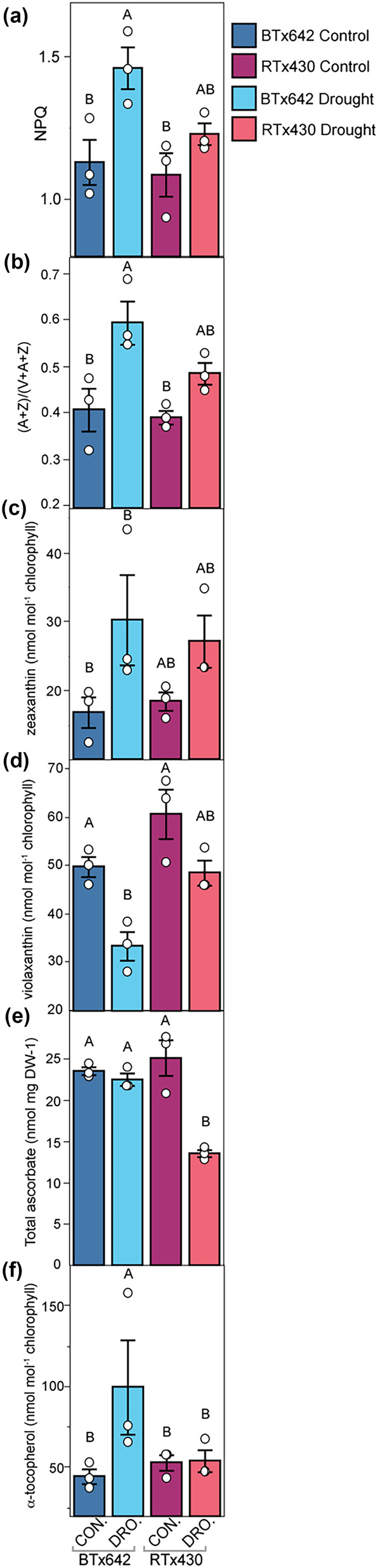
Photoprotective response to terminal drought stress. From the D4 timepoints, (a) non‐photochemical quenching (NPQ) measured at mid‐afternoon, (b) epoxidation state of the Violaxanthin + Antheraxanthin + Zeaxanthin (VAZ) pool measured as (A + Z)/(V + A + Z), (c) zeaxanthin, (d) violaxanthin, (e) total ascorbate levels, and (f) α‐tocopherol levels. (c–g) Data are from leaf samples collected in the mid‐afternoon. Mean values ± standard errors (*n* = 3 plots) with mean values for each individual plot displayed as dots (white). BTx642 control (dark blue), BTx642 drought (light blue), RTx430 control (purple), and RTx430 drought (pink). Significant differences as measured by a two‐tailed *t* test for control versus treatment pairs are indicated by asterisks (* < .05). Mean values that share the same letters are not statistically different, and those that do not share the same letters are statistically different based on one‐way analysis of variance (ANOVA) and post hoc Tukey–Kramer honest significant difference (HSD) tests.

**FIGURE 6:**
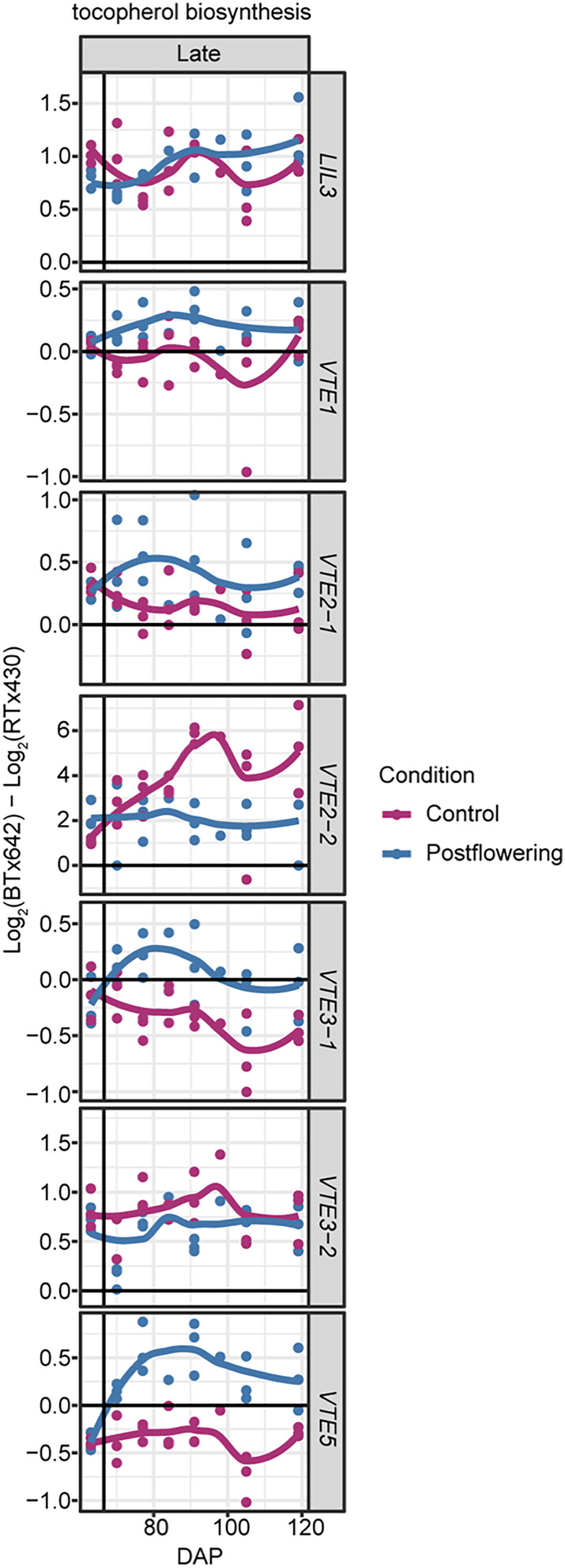
Log_2_ fold change in transcript abundance for tocopherol biosynthesis genes across genotypes (BTx642 versus RTx430) in leaf tissue for drought versus control: Sobic.004G024600 (*LIL3*), Sobic.004G125800 (*VTE1*), Sobic.010G215600 (*VTE2‐1*), Sobic.010G207900 (*VTE2‐2*), Sobic.008G171300 (*VTE3‐1*), Sobic.008G171000 (*VTE3‐2*), and Sobic.006G260800 (*VTE5*). Log_2_ (BTx642 control/RTx430 control) in dark blue and log_2_ (BTx642 post‐flowering drought/RTx430 post‐flowering drought) in purple.

NPQ was induced specifically in droughted BTx642 (Figure 5a). Supporting this genotype‐specific induction of NPQ, the de‐epoxidation state of the xanthophyll pool was also specifically higher in droughted BTx642 relative to the control conditions (Figure 5b–d). NPQ and pigment measurements were performed exclusively on green leaf tissue to prevent superficially low NPQ values in RTx430 due to the higher percentage of senesced tissue in RTx430 droughted plots. Beyond NPQ, higher total ascorbate levels were maintained in droughted BTx642 in contrast to RTx430 (Figure 5e, S2). Further, the chloroplast‐localized antioxidant α‐tocopherol and the epidermis‐enriched photoprotective flavonoid, rutin, were specifically induced in droughted BTx642 (Figure 5f, S2). Consistent with the induction of α‐tocopherol specifically in droughted BTx642, higher transcript levels were observed for several genes involved in tocopherol biosynthesis, such as Sobic.004G024600 (*LIL3*), Sobic.010G207900 (*VTE2‐2*), and Sobic.006G260800 (*VTE5*), in droughted BTx642 relative to droughted RTx430 (Figure 6).

## 4 DISCUSSION

Responses to drought differ dramatically across the lifecycle stage of the plant. Post‐flowering drought has been intensively studied in an agronomic context; however, molecular pathways for post‐flowering drought tolerance have not historically been given the same attention as drought responses in early plant lifecycle stages. Further, the plant's molecular response to drought can fundamentally differ between field‐droughted and greenhouse‐droughted plants (Varoquaux et al., 2019). We have combined in‐field physiological analysis with transcriptomic and metabolomic analysis on field‐droughted plants grown in replicated plots for two sorghum genotypes, BTx642 and RTx430. Our goals were twofold. First, a time‐course dataset of leaf gas exchange data was collected from both genotypes in control and droughted plots in parallel with sample collection for metabolomic analysis to determine which metabolites may act as potential regulators of photosynthetic activity in post‐flowering droughted sorghum. Second, we wanted to test whether the genotypes of sorghum that most proficiently maintain photosynthetic activity in drought (i.e., the functional “stay green” phenotype) exhibit stronger induction of photoprotective mechanisms relative to non‐functional stay greens.

Highlighting the strong drought tolerance of sorghum, both RTx430 and BTx642 maintained *A*_*n*_ values on par with control plants in the morning measurements (9:30 to 11:00) throughout the grain filling period despite terminal water deprivation for 40 days (Figures 1, 2). The depression of *A*_*n*_ in mid‐afternoon measurements in droughted plants was tightly coupled to *g*_*s*_ (Figure 2). Both RTx430 and BTx642 can be considered drought‐tolerant genotypes given their yield data, yet their drought tolerance strategies diverge (Figure 2, Tables 2 and S2). For instance, BTx642 maintained more closed stomata in control plots relative to RTx430, and thus, the net decline in *A*_*n*_ in mid‐afternoon measurements was sharper for droughted RTx430 relative to droughted BTx642 (Table 2). We note that this is a clear example of how high photosynthetic rates under well‐watered conditions are not predictive of higher photosynthetic rates in drought conditions in the absence of other beneficial traits (Blum, 2009; Harris et al., 2007).

### 4.1 Galactinol and the metabolic control of *g*_*s*_ in post‐flowering drought

Whereas the abundance of many lipids and metabolites responded to post‐flowering drought, the response of only a handful of metabolites responded to post‐flowering drought in a shared pattern in BTx642 and RTx430 (Figures 3 and S2, Table S4). For instance, while both genotypes increased the abundance of leaf osmolytes, consistent with the measured increase in leaf osmotic potential, there was little overlap between the specific osmoprotective molecules elevated in each genotype (Figures 1E and S2). It also worth noting that metabolites important for the drought response in sorghum seedlings, such as proline and ABA, were not significantly increased by drought in post‐flowering droughted leaf tissue (Figure S2).

The abundance of four metabolites correlated with *g*_*s*_ and *A*_*n*_ activity (Table S1). Two of these metabolites, fumarate and its isomer maleate, were inversely correlated with *g*_*s*_, meaning that their abundance increased with the drought (Figure 3, Table S1). Fumarate levels have been previously suggested to control stomatal aperture based on work in *Arabidopsis thaliana*, and the role of this metabolite in regulating *g*_*s*_ in drought has been speculated upon (Araújo et al., 2011; Ferreira et al., 2021). However, the linear curve fitting for both fumarate and maleate levels and *g*_*s*_ was not robust as evidenced by the non‐Normal distribution of the curve fitting residuals (Table S1). Strong increases in fumarate/maleate abundance were not observed until D4, whereas *g*_*s*_ already began responding to the drought at the D2 and D3 sampling dates. We conclude that because fumarate/maleate levels appear to change after *g*_*s*_ levels have already responded to the drought, they are likely to be responding to drought signals rather than acting as signaling molecules that repress stomatal opening.

In contrast, galactinol had a robust positive correlation with *g*_*s*_ and *A*_*n*_ for data collected from replicated field plots (Figure 3, Table S1). Galactinol is synthesized by galactinol synthase (GolS1 and GolS2, Figure 3). Genetic evidence for the role of GolS2 in drought tolerance was first discovered in *A. thaliana*, where overexpression of this enzyme reduced transpiration rates and improved drought tolerance (Taji et al., 2002). Both galactinol and raffinose levels were increased hundreds‐of‐fold in leaf tissue in these transgenic lines, and thus, it was concluded that improved drought tolerance was likely to be a consequence of the massively increased osmolytes and not the effect on transpiration. Overexpression of GolS2 in rice produced similarly improved drought tolerance under field conditions; however, the effect on transpiration and *g*_*s*_ was not quantified in this study (Selvaraj et al., 2017). In maize, the connection between high galactinol levels and increased drought tolerance was challenged by the discovery that the drought‐susceptible phenotype of *zmrafs‐1* was caused by a loss‐of‐mutation in a galactinol hydrolyase that caused the over‐accumulation of galactinol (Li et al., 2020). Remarkably, overexpression of ZmRAFS in *A. thaliana* both increased drought tolerance while significantly decreasing galactinol and raffinose levels, contradicting the conclusion that GolS2 overexpression improved drought tolerance via increasing osmolyte abundance (Li et al., 2020). Again, *g*_*s*_ was not measured in that work.

In our work, galactinol levels decreased in concert with *g*_*s*_ (Figure 3). Raffinose levels were also lower at the D4 timepoint in both genotypes (Figure S2, Table S4). Both transcript levels for GolS2 and SbRAFS responded to post‐flowering drought in a manner that is consistent with the gradually declining galactinol levels in droughted plants (Figure 3). GolS2 levels declined gradually over the post‐flowering drought time‐course, potentially slowly reducing the supply of galactinol, whereas the SbRAFS transcript was induced throughout the post‐flowering drought, perhaps accelerating the hydrolysis of galactinol. In future work, link galactinol levels directly to control of *g*_*s*_ activity in post‐flowering drought, it would be useful to develop a loss‐of‐function sorghum mutant in SbRAFS, as well as an overexpression line for this gene. Based on our data, the *sbrafs* mutant should have high galactinol levels, high *g*_*s*_, and low post‐flowering drought tolerance, whereas overexpression might produce the inverse effects.

### 4.2 Photoprotection and the functional stay‐green trait in sorghum

BTx642 is a stay‐green sorghum genotype, a characteristic easily observed visually in our post‐flowering droughted plots (Figure 4). While the characteristics underlying the functional stay‐green genotype in sorghum have been a subject of continuous research, the role that photoprotective responses may have in limiting photooxidative damage and thereby minimizing the extent of drought‐induced early leaf senescence in stay‐greens has not been investigated.

The role of photoprotection in preventing drought‐induced leaf senescence is well‐established (Challabathula et al., 2018; Demmig‐Adams & Adams, 2006; Munné‐Bosch et al., 2001; Munné‐Bosch & Peñuelas, 2003; Murchie et al., 1999). In this study, in the later stages of drought, F_v_/F_m_ and green leaf area declined to a greater extent in RTx430 (Figure 2h, Table 1). One stimulating factor increasing leaf senescence in post‐flowering drought may be the demand to recycle leaf N content to support the N demand of developing seeds (Borrell et al., 2001). Both leaf C/N and *N*_*org*_ levels were, in fact, reduced by drought in both genotypes, but no genotype or genotype × treatment effect was found for these parameters (Table 2). In contrast, the loss of green leaf area in droughted plots had a strong genotype × environment effect. Thus, leaf *N*_*org*_ level was decreased by post‐flowering drought, but this effect did not explain the difference in green leaf area or the maintenance of photosynthetic activity in drought (*A*_*n*_) between the two genotypes.

The BTx642 genotype more strongly induced a suite of photoprotective responses, supporting the hypothesis that the stay‐green BTx642 would have a stronger photoprotective response in post‐flowering drought (Figures 5 and S2). These included genotype‐specific drought induction of NPQ and photoprotective molecules (e.g., α‐tocopherol [Figure 5] and rutin [Figure S2]) and maintenance of high ascorbate pool size (Figures 5 and S2). The higher abundance of transcripts for tocopherol biosynthetic genes in the stay‐green genotype BTx642 suggests that boosting tocopherol levels in RTx430 via over‐expression of tocopherol biosynthesis enzymes may be one avenue to inhibit drought‐induced early leaf senescence in sorghum (Figure 6) (Liu et al., 2008; Zhan et al., 2019). A second avenue to improve stay‐green capacity in RTx430 could involve increasing NPQ capacity by over‐expression of the NPQ regulator *PSBS* during post‐flowering drought or by supporting a more de‐epoxidized xanthophyll cycle pool for NPQ (Głowacka et al., 2018).

As climate change constrains agricultural productivity in the coming decades, conferring functional post‐flowering drought tolerance to drought‐susceptible genotypes can improve yields with limited water inputs. Here, we have applied metabolomic, transcriptomic, and physiological measurements across time to post‐flowering droughted, field grown sorghum plants in a RCBD. By bringing together these different techniques, we were able to discover an unanticipated correlation between foliar galactinol levels and stomatal conductance in post‐flowering drought. This finding is further supported by features of our transcriptomic data, as well as previously published findings, as discussed above. We also find stronger induction of photoprotective mechanisms in the stay‐green BTx642 genotype, providing a new type of explanation for the mechanisms underlying the functional stay‐green phenotype. Given the importance that improved post‐flowering drought tolerance may have for agriculture in a water‐scarce world, we hope that future work will test these findings, currently resting on correlative data, through generating mutants that alter foliar galactinol levels and manipulating photoprotective responses to directly test their role in the functional stay‐green phenotype.

## AUTHOR CONTRIBUTIONS

CRB, KKN, PGL, JD, RBH, and KK designed and planned the research. CRB and DP conducted experiments, collected field samples, and analyzed data. LGC, OD, and AK conducted experiments and collected/processed field samples. RBH, BJC, AB, JYL, YMK, JEK, KJB, and VP conducted experiments and analyzed data. CA, JP, and JS collected/processed field samples. CRB wrote the manuscript with contributions from BJC, AB, JYL, and YMK. The manuscript was edited by KKN, JD, PGL, DP, and AK. OD and AK contributed equally to this work. CA, JP, and JS contributed equally to this work. AB, JYL, and YMK contributed equally to this work.

## CONFLICT OF INTEREST STATEMENT

The authors declare that they have no conflict of interest, financial or otherwise, that influenced this manuscript.

## PEER REVIEW

The peer review history for this article is available in the Supporting Information for this article.

## Supporting information


**Data S1.** Peer Review.

## Data Availability

The data that support the findings of this study are available from the corresponding author upon reasonable request.
