# Supplementary material for: Metabolomic, photoprotective, and photosynthetic acclimatory responses to post‐flowering drought in sorghum
Source: Plant Direct. 2023 Nov 13;7(11):e545. doi: 10.1002/pld3.545 (PMC10641490; doi:10.1002/pld3.545)
Supplement: Supplementary file 1 — Data S1. Peer Review. [file PLD3-7-e545-s001.pdf]

Plant Direct
Open Access

Metabolomic, photoprotective, and photosynthetic acclimatory responses to post-flowering drought in sorghum.

Christopher R. Baker[1*], Dhruv Patel-Tupper[2], Benjamin J. Cole[3], Lindsey G. Ching[2], Oliver Dautermann[2], Armen C. Kelikian[2], Cayci Allison[4], Julie Pedraza[4], Julie Sievert[4], Aivett Bilbao[5], Joon-Yong Lee[6], Young-Mo Kim[6], Jennifer E. Kyle[6], Kent J. Bloodsworth[6], Vanessa Paurus[6], Kim K. Hixson[5], Robert Hutmacher[7], Jeffery Dahlberg[4], Peggy G. Lemaux[2], Krishna K. Niyogi[1,8]

DECISION LETTER- ROUND 1

September 28, 2023

Christopher Baker
University of California, Berkeley
Berkeley, California

RE: Metabolomic, photoprotective, and photosynthetic acclimatory responses to post-flowering drought in sorghum

Dear Dr. Baker:

Thank you for submitting to Plant Direct. In light of the reviewer's comments, some minor revisions are needed before the paper can be accepted for publication in Plant Direct.

Please view the reviewers' comments below and use the suggestions as a guide while you work on your revision.

When uploading the revised version of this article, please be sure to include the following:

-A word document that contains your response to the reviewers. You should respond to each reviewer comment and note the changes made to the manuscript. If you do not agree with a reviewer's comment and choose not to make a suggested revision, please explain why. Please try to provide as complete an answer as possible to each reviewer's criticisms.
-A tracked changes document with each change highlighted
- A clean version of the latest version of the manuscript

To upload your revision, please click the link below.
https://plantdirect.msubmit.net/cgi-bin/main.plex?el=A2Lr4cJ2A3oLx5I7A9ftda6p9eMrhXlTYhZrBNKNIwAZ

In order to provide as timely a service as possible, we ask that your revision is resubmitted within two months after receipt of this request. If an extension is needed, please send a request, along with a brief explanation, to the editorial office at plantdirect@wiley.com .

Please note that, in addition to publishing reviewer comments, the author's responses to review comments will also be published alongside the final version of the paper. If you would not like the author's responses to be published, please contact the editorial office at plantdirect@wiley.com .

Thank you very much for giving us an opportunity to review your work. I look forward to receiving the next version.

Sincerely,

Feng Chen

Editor, Plant Direct

-- Reviewer comments:
Reviewer #1:

The manuscript, "Metabolomic, photoprotective, and photosynthetic acclimatory responses to post-flowering drought in sorghum," describes field research that linked physiological mechanisms to metabolites and gene expression in sorghum. I really liked this approach linking physiology and molecular biology and had no major concerns with the well-reported work. I had some suggestions for describing the layout more clearly, the statistical method, and which roots were sampled.

I do wonder if the authors have considered multivariate techniques like PCA to combine the traits. In my mind this is usually lacking in such studies but could be a very powerful approach to gain more understanding in such valuable and hard to acquire datasets. Another example would be multiple regression. For example, a single metabolite had a decent correlation to gs, but what about multiple metabolites? This could explain some of the variation. Just some thoughts for the authors to consider that could make the study more compelling and increase their understanding, not a requirement.

155: If not mentioned elsewhere, how many replications? How was the irrigation applied? Randomized design? A diagram of the layout would be useful. - I see sup figure 1 does this, don't think it was referenced. Looks like a Randomized Complete Block Design (RBCD) with three replications which should be stated, and the statistics should be appropriate for the block design. Genotype and water levels are factorial and randomly applied.

161: is 'field hydration maximum' meant to refer to 'field capacity'? The latter is the more common soil science term used.

221: A little more detail on root sampling is needed. A reader shouldn't have to dig up another paper

to know which roots were sampled from where. Just like leaves were explained down to the specific leaf. For maize, it should be possible to state if they were nodal, seminal, or primary (tap). If nodal, crown or brace? What was the approximate diameter? Did it include a parent root and laterals? Were root tips present? These are some of the parameters that would help describe such a root sample. If from a core, what diameter core and what depth range.

258: Giving the software of stats is less important than the model. The model should be appropriate to the RBCD design. You will get off easy if nobody complains about how non independent time-series measures were handled.

260: how is Ci constrained mathematically? Give the possible range.

371: mentions two way ANOVA so that is what was needed in the stats methods.

500: I like that the discussion seems reasonable and careful. I think a few sentences before the conclusion would be useful giving some of the limitations of the study and how more clear links could be made between observed gene expression, metabolites, and physiological functions.

Fig 3C. I really like this direct test of the metabolite versus conductance. The relation isn't perfect but it's compelling that they are somehow related.

DECISION LETTER- ROUND 2

October 12, 2023
christopher baker
University of California, Berkeley
Berkeley, California

MSID: 2023-01260R1
MS TITLE: Metabolomic, photoprotective, and photosynthetic acclimatory responses to post-flowering drought in sorghum

Dear Dr. christopher baker:

I am pleased to inform you that your manuscript "Metabolomic, photoprotective, and photosynthetic acclimatory responses to post-flowering drought in sorghum" has been accepted for publication in Plant Direct.

Your article will appear online in the next available issue of Plant Direct. To ensure your article gets published as quickly as possible, please pay attention to the steps detailed below. We have found that most of the delays happen at this stage, especially at the payment stage, so please respond as quickly as possible when prompted.

License Agreement: Once your article has been accepted it will move to Production and undergo admin and file checking - you may receive an email with any queries we have at this stage. When all required items are received by the publisher and queries resolved, the corresponding author will

receive an email from Wiley's Author Services system which will ask them to log on at https://authorservices.wiley.com/bauthor and will present them with the appropriate license for completion. Your article cannot be published until both the signed license agreement and payment of the article fee have been received.

Payment of the Open Access Article Publication Fee: All articles published in Plant Direct are fully open access: immediately and freely available to read, download and share. Plant Direct charges a publication fee to cover the publication costs. The corresponding author for this manuscript should have already received a quote from the payments team (cs-openaccess@wiley.com) with the estimated article publication fee; please email cs-openaccess@wiley.com if this has not been received. The corresponding author should log on to the Wiley Author Services site, where the publication fee can be paid by credit card or an invoice. Pro Forma can also be requested. Payment of the publication charge must be received before the article will be published online.

Proofs: You will have the opportunity to look over your paper once more when you receive the author proofs for your article. The proofs will be with you in approximately two weeks. Please note that, in addition to publishing reviewer comments, the author's responses to review comments will also be published alongside the final version of the paper. If you would not like the author's responses to be published, please contact the editorial office at plantdirect@wiley.com .

Promotion of your article: You can help your research get the attention it deserves! Check out Wiley's free Promotion Guide for best-practice recommendations for promoting your work at www.wileyauthors.com/eeo/guide. And learn more about Wiley Editing Services which offers professional video, design, and writing services to create shareable video abstracts, infographics, conference posters, lay summaries, and research news stories for your research at www.wileyauthors.com/eeo/promotion.

Thank you again for your contribution to Plant Direct. If you have any questions, feel free to contact the editorial office at plantdirect@wiley.com .

Sincerely,

Feng Chen

Editor, Plant Direct

AUTHORS' RESPONSE

-- Reviewer comments:
Reviewer #1:

The manuscript, "Metabolomic, photoprotective, and photosynthetic acclimatory responses to post-flowering drought in sorghum," describes field research that linked physiological mechanisms to metabolites and gene expression in sorghum. I really liked this approach linking physiology and molecular biology and had no major concerns with the well-reported work. I had some suggestions for describing the layout more clearly, the statistical method, and which roots were sampled.

I do wonder if the authors have considered multivariate techniques like PCA to combine the traits. In my mind this is usually lacking in such studies but could be a very powerful approach to gain more understanding in such valuable and hard to acquire datasets. Another example would be multiple regression. For example, a single metabolite had a decent correlation to gs, but what about multiple metabolites? This could explain some of the variation. Just some thoughts for the authors to consider that could make the study more compelling and increase their understanding, not a requirement.

We agree that multivariate approaches can provide powerful insights to such a study. As the reviewer points out, it would be quite interesting to see if some of the noise in the galactinol correlation with stomatal conductance could be explained by a second metabolite. However, it is the opinion of authors that the paper is already quite data heavy, and it is our strong opinion that such an analysis would distract from the primary findings of the paper. Thus, we have chosen not to include this further analysis in the revised manuscript and have incorporated this suggested analysis in the end of the Discussion section:

> *Given the importance that improved post-flowering drought tolerance may have for agricultural in a water scarce world, we hope that future work will test these findings, currently resting on correlative data, through generating mutants that alter foliar galactinol levels and manipulating photoprotective responses to directly assess their role in post-flowering drought tolerance in field conditions. Even without further data collection, additional insights may be gleaned by further analyzing the rich datasets described here via principal component analysis (PCA) or multiple regression analysis of multiple metabolites with the measured physiological parameters.*

155: If not mentioned elsewhere, how many replications? How was the irrigation applied? Randomized design? A diagram of the layout would be useful. - I see sup figure 1 does this, don't think it was referenced. Looks like a Randomized Complete Block Design (RBCD) with three replications which should be stated, and the statistics should be appropriate for the block design. Genotype and water levels are factorial and randomly applied.

The relevant text has been updated include more detail on replication, irrigation, and field layout. We have also sought to highlight these details more clearly by breaking this section into two paragraphs with one paragraph dedicated specifically to irrigation. We have also changed the subsection header to indicate that irrigation information can be found in this section of the Materials and Methods:

> *Sorghum genotypes BTx642 and RTx430 were grown in Parlier, CA (36.6008°N, 119.5109°W) in 2019 in a Hanford sandy loam soil (pH = 7.37) with a silky substratum in 0.071 hectare (ha) plots of ten rows each. Plots were arranged in a Randomized Complete Block Design (RCBD) with three replications of each genotype and water regime (Figure S1). Two watering conditions were used on plots: (1) control, consisting of weekly watering, based on*

evapotranspiration, five days prior to sampling dates, with the first irrigation starting 18 days after planting (DAP) and continuing until 123 DAP and (2) post-flowering drought, consisting of regular irrigation up through and including irrigation at 65 DAP – at which point over 50% of the plants flowered (anthesis) – with terminal water deprivation from that point onwards (Figure 1). Pre-planting irrigation was performed for all plots such that the upper 122 cm of soil would have been refilled to the field capacity. Following that, plots receiving water were irrigated at seven-day intervals using drip irrigation lines placed on the soil surface of each furrow.

All irrigated plots received volumes of water equal to 100% of the average weekly calculated crop evapotranspiration for the 7-day period before irrigation. Surface drip lines were used for irrigation to provide accurate water application amounts and a high level of water application uniformity. Irrigation once per week in plots is comparable to sorghum farming irrigation practices in the Western United States. Additionally, providing equal water volume to all irrigated plots prevents a scenario where genotypic differences in evapotranspiration rates lead to a difference in total water volume supplied to specific plots. For greater details on crop evapotranspiration and irrigation management, see supplemental materials and Xu et al. (Xu et al., 2018).

161: is 'field hydration maximum' meant to refer to 'field capacity'? The latter is the more common soil science term used.

The reviewer is correct. This term has been changed to 'field capacity'.

221: A little more detail on root sampling is needed. A reader shouldn't have to dig up another paper to know which roots were sampled from where. Just like leaves were explained down to the specific leaf. For maize, it should be possible to state if they were nodal, seminal, or primary (tap). If nodal, crown or brace? What was the approximate diameter? Did it include a parent root and laterals? Were root tips present? These are some of the parameters that would help describe such a root sample. If from a core, what diameter core and what depth range.

We have added this information as requested. The relevant text has been updated to:

Plant samples were collected manually on the same days as physiological measurements with root systems to a depth of approximately 30 cm. Three plants from each plot were collected, and the uppermost three leaves, stems (below the peduncle and above the node for the next leaf below), and roots were harvested to create a single leaf, stem, and root sample for each plot for each timepoint. Root tissue was collected as previously described (Xu et al., 2018). Root tissue collection avoided brace roots and consisted almost entirely of mature, differentiated roots, avoiding root tips to make this sample more comparable to the mature leaf tissue. After collection, roots were vortexed in epiphyte removal buffer (0.75% KH_2PO_4, 0.95% K_2HPO_4, 1% Triton X-100 in ddH2O; filter sterilized at 0.2 µm) for 5 min.

258: Giving the software of stats is less important than the model. The model should be appropriate to the RBCD design. You will get off easy if nobody complains about how non independent time-series measures were handled.

The relevant text has been updated to:

> *All statistical analyses were performed using JMP Pro 16 software (JMP, Cary, NC, USA) and ANOVA used to analyze the effects of treatment and genotype, appropriate for the RCBD field layout.*

260: how is Ci constrained mathematically? Give the possible range.

Values below 0 are physiological impossible. The relevant text has been updated to read:

> *Prior to the analysis of gas exchange values, six measurements (out of the 462 measurements taken) with physiologically impossible C_i values (C_i values < 0 mMol CO_2 mol^{-1} air) were removed from our datasets and attributed to either machine or user error.*

371: mentions two way ANOVA so that is what was needed in the stats methods.

See above.

500: I like that the discussion seems reasonable and careful. I think a few sentences before the conclusion would be useful giving some of the limitations of the study and how more clear links could be made between observed gene expression, metabolites, and physiological functions.

We thank the reviewer for these kind words. The closing paragraph has been rewritten to be more specific and highlight that the data in this study is ultimately correlative, and work that tests causation will be required in the future:

> *As climate change constrains agricultural productivity in the coming decades, conferring functional post-flowering drought tolerance to drought-susceptible genotypes can improve yields with limited water inputs. Here, we have applied metabolomic, transcriptomic, and physiological measurements across time to post-flowering droughted, field grown sorghum plants in a Randomized Complete Block Design. By bringing together these different techniques, we were able to discover an unanticipated correlation between foliar galactinol levels and stomatal conductance, and thereby net CO_2 assimilation rates as well, in post-flowering drought. This finding is further supported by features of our transcriptomic data, as*

well as previously published findings, as discussed above. We also find stronger induction of photoprotective mechanisms in the stay-green BTx642 genotype, providing a new type of explanation for the mechanisms underlying the functional stay-green phenotype. Given the importance that improved post-flowering drought tolerance may have for agriculture in a water-scarce world, we hope that future work will test these findings, currently resting on correlative data, through generating mutants that alter foliar galactinol levels and manipulating photoprotective responses to directly assess their role in post-flowering drought tolerance in field conditions.

Fig 3C. I really like this direct test of the metabolite versus conductance. The relation isn't perfect but it's compelling that they are somehow related.

We thank the reviewer for these kind words.
